# GO Explorer: A gene-ontology tool to aid in the interpretation of shotgun proteomics data

**DOI:** 10.1186/1477-5956-7-6

**Published:** 2009-02-24

**Authors:** Paulo C Carvalho, Juliana SG Fischer, Emily I Chen, Gilberto B Domont, Maria GC Carvalho, Wim M Degrave, John R Yates, Valmir C Barbosa

**Affiliations:** 1 Systems Engineering and Computer Science Program, Federal University of Rio de Janeiro, Brazil; 2 Department of Chemical Physiology, The Scripps Research Institute, La Jolla, USA; 3 Chemistry Institute, Federal University of Rio de Janeiro, and Rio de Janeiro Proteomics Network, Rio de Janeiro, Brazil; 4 Carlos Chagas Filho Biophysics Institute, Federal University of Rio de Janeiro, Rio de Janeiro, Brazil; 5 Oswaldo Cruz Institute, Laboratory for Functional Genomics and Bioinformatics, Rio de Janeiro, Brazil; 6 Department of Pharmacological Sciences, Stony Brook University, Stony Brook, NY, USA

## Abstract

**Background:**

Spectral counting is a shotgun proteomics approach comprising the identification and relative quantitation of thousands of proteins in complex mixtures. However, this strategy generates bewildering amounts of data whose biological interpretation is a challenge.

**Results:**

Here we present a new algorithm, termed GO Explorer (GOEx), that leverages the gene ontology (GO) to aid in the interpretation of proteomic data. GOEx stands out because it combines data from protein fold changes with GO over-representation statistics to help draw conclusions. Moreover, it is tightly integrated within the PatternLab for Proteomics project and, thus, lies within a complete computational environment that provides parsers and pattern recognition tools designed for spectral counting. GOEx offers three independent methods to query data: an interactive directed acyclic graph, a specialist mode where key words can be searched, and an automatic search. Its usefulness is demonstrated by applying it to help interpret the effects of perillyl alcohol, a natural chemotherapeutic agent, on glioblastoma multiform cell lines (A172). We used a new multi-surfactant shotgun proteomic strategy and identified more than 2600 proteins; GOEx pinpointed key sets of differentially expressed proteins related to cell cycle, alcohol catabolism, the Ras pathway, apoptosis, and stress response, to name a few.

**Conclusion:**

GOEx facilitates organism-specific studies by leveraging GO and providing a rich graphical user interface. It is a simple to use tool, specialized for biologists who wish to analyze spectral counting data from shotgun proteomics. GOEx is available at .

## Background

Shotgun proteomics is a strategy capable of identifying thousands of proteins in complex mixtures. Its methodology comprises the pre-digestion of proteins followed by peptide separation, fragmentation in a mass spectrometer, and database search [1,2]. Multi-dimensional Protein Identification Technology (MudPIT) is a shotgun proteomics technique capable of identifying thousands of proteins in proteolytically digested complex mixtures [2,3]. MudPIT separates peptides according to two independent physicochemical properties using two-dimensional liquid chromatography (LC/LC) online with the ion source of a mass spectrometer. This separation relies on columns of strong cation exchange (SCX) and reversed phase (RP) material, back to back, inside fused silica capillaries. The chromatography proceeds in cycles, each of which consists of increasing salt concentration to "bump" peptides off the SCX followed by a hydrophobic gradient to progressively elute peptides from the RP into the ion source. This process identifies mixture components by tandem mass spectrometry (MS/MS). Relative protein quantitation can be obtained through tandem mass spectral features (e.g., peptide hits, protein sequence coverage, spectral counts) [1,3-5]. For example, Liu *et al*. demonstrated that the number of tandem mass spectra obtained for each protein, or "spectral count", linearly correlates with its abundance in a mixture by two orders of magnitude [6]. Currently, spectral counting is a widely adopted approach to characterize different states of biological systems according to protein expression differences.

Acquiring a holistic understanding over a large set of proteins is not a simple task, but first insights can be obtained by searching the Gene Ontology (GO) [7] annotations for over-represented terms. GO is a standard for functional annotation and consists of structured and controlled vocabularies to classify terms into the following root categories (namespaces): molecular function, biological processes, and cellular components. Its structure follows that of a directed acyclic graph (DAG); each term is a more specific child of one or more parents (i.e., directed edges point in the direction of increasing specificity). In this way, a convention named *true path rule *states that whenever a gene is annotated with a term, it is also implicitly annotated with all (less specific) ancestors of that term.

Currently, there are several GO-based tools; some examples are: DAVID [8], GOMiner [9], and GoFish [10]. We refer the reader to  for a more comprehensive listing. Even though such tools are frequently used to analyze microarray data, the ones specific for proteomics amount to very few [11]. Moreover, most existing GO-based tools for proteomics overlook expression fold changes and, as far as we know, are not specialized in directly handling data from differential proteomic spectral counting experiments. One exception with relation to the use of fold changes is GESA (Gene Enrichment Analysis) [12], which ranks genes according to expression quantitation data and then correlates them to search for enriched GO terms. However, limiting the search to enriched terms can hide very subtle results elucidated by individual proteins. In this respect, we note that GOEx provides several exploratory methods that are not bound to finding terms that are necessarily enriched but could be related even to one single protein.

In this work we present a new GO-based tool, named GO Explorer (GOEx), which is optimized to work with spectral counting data from shotgun proteomics. This is achieved, in part, because GOEx is natively integrated into the PatternLab for Proteomics project [13] so it leverages existing parsers, data normalization, and feature selection algorithms designed to work with spectral counts. GOEx allows one to explore data using several new approaches as described in the Implementation section.

We demonstrate GOEx by using proteomic data acquired from human glioblastoma multiform (GBM) cell lines (A172) both before and after applying perillyl alcohol (POH) to their medium. Briefly, POH is a naturally occurring monoterpene found in lavender, cherries, and mint, and is a promising chemotherapeutic agent. In human cancer cells, POH has shown cytostatic and cytotoxic effects [14-16], inducing apoptosis on lung [17], leukemia [18], prostate [19], and breast [20] cancer cell lines. POH is also under evaluation in several clinical trials, including an ongoing phase I comprising GBM patients treated by intranasal delivery that has shown promising results [21].

## Experimental: preparation of the A172-POH dataset

### Materials

Invitrosol™ and RapiGest™ SF acid-labile surfactant were purchased from Invitrogen (Carlsbad, CA) and Waters Corp. (Milford, MA), respectively. PPS Slient surfactant was provided by Dr. Norris from Protein Discovery, Inc. (Knoxville, TN). The proteases endoproteinase Lys-C and trypsin (modified, sequencing grade) were obtained from Roche. Human malignant glioma cells (A172) were obtained from the American Type Culture Collection. POH and other laboratory reagents were purchased from Sigma-Aldrich (St. Louis, MO), unless noted otherwise.

### Cell culture and POH treatment

The A172 cells were grown as monolayers in 25 cm^2 ^tissue culture flasks in Dulbecco's modified Eagle medium supplemented with 0.2 mM non-essential amino acids, 10% fetal calf serum, penicillin (60 *μ*g/mL), streptomycin (100 *μ*g/mL), and amphotericin B (fungizone, 2.5 mg/mL). For sub-cultivations, confluent monolayers were gently washed with phosphate-buffered saline (PBS 1×) pH 7.2, and after short trypsinization the cells were suspended in culture medium. Three subcultures were treated with 1.8 mM POH (Sigma-Aldrich, 96%) during 1.5 h and three other subcultures received no POH treatment; the cellular morphology analyzed by an optical phase-contrast microscope (Zeiss Axioplan, Thornwood, NY) and the cells were photographed. The medium from all cultures was discarded and the cells were rinsed twice with PBS (1×). The cells were detached from the flask by exposing them during 2 min in a solution of 0.25% trypsin-EDTA (1×). Then the cells were re-suspended in the medium and a pellet was obtained by centrifugation during 10 min at 500 RCF. This procedure was performed three times. Proteins were extracted from the cell pellets using the total protein extraction kit from Biochain (Hayward, CA) according to manufacturer's instructions.

### Protein solubilization with MS-compatible detergents and trypsin digestion

Each protein pellet was re-suspended, independently, with one of the following MS-compatible detergents: 5 *μ*L of Invitrosol (5× stock), RapiGest SF (1% stock), or 10 *μ*L of PPS (1% stock). We recall that these detergents are called MS-compatible because they do not interfere with the mass spectral acquisition, increase proteolytic efficiency, and peptide and protein identifications in complex protein mixtures analyzed by shotgun proteomics [22]. The concentration of each detergent used in this study was determined based on the maximum recommended concentration suggested by the manufacturers. Then the proteins were incubated at 60°C for 5 min and completed with solvent (PPS reconstituted in the same buffer, RapiGest reconstituted in 50 mM ammonium bicarbonate, Invitrosol is already sold in solution) to a 50 *μ*L final volume. All samples were sonicated for 2 h in a water bath and digested with trypsin (1:50) for 16 h at 37°C.

### Post-digestion

Following digestion, all reactions were acidified with 90% (v/v) formic acid (2% final) to stop the proteolysis. Samples with RapiGest SF and PPS were acidified and incubated at 37°C for additional 4 h to facilitate the hydrolysis of the detergents. Then samples were centrifuged for 30 min at 14,000 rpm to remove insoluble material. The soluble peptide mixtures were collected, dried by a Speed Vac, reconstituted in 10 *μ*L of buffer A (95% H_2_O (v/v), 5% acetonitrile (v/v), and 0.1% formic acid (v/v)), and analyzed by MudPIT[1].

### Protein identification by MudPIT

Approximately 70 *μ*g of the digested peptide mixture were loaded onto a biphasic (strong cation exchange/reversed phase) capillary column and washed with a buffer containing 5% acetonitrile, 0.1% formic acid diluted in HPLC grade water. The two-dimensional liquid chromatography separation and tandem mass spectrometry conditions were as described by Washburn *et al*. [1]. The flow rate at the tip of the biphasic column was 300 nL/min when the mobile phase composition was 95% H_2_O, 5% acetonitrile, and 0.1% formic acid. The ion trap mass spectrometer, Finnigan LCQ Deca XP (Thermo Finnigan, San Jose, CA), was set to the data-dependent acquisition mode with dynamic exclusion turned on. One MS survey scan was followed by four MS/MS scans and 12 salt steps were performed. Mass spectrometer scan functions and HPLC solvent gradients were controlled by the Xcalibur data system (Thermo Finnigan, San Jose, CA).

Tandem mass spectra were extracted from the raw files, and a binary classifier, previously trained on a manually validated dataset, was used to remove the low-quality MS/MS spectra [23]. The remaining spectra were searched against the *Homo sapiens *protein plus common contaminant proteins; all sequences were downloaded as FASTA-formatted from the EBI-IPI protein database (database version 3.23, released on November 2, 2006) [24]. To calculate confidence levels and false-positive rates, a decoy database that contained the reverse sequences of the original dataset appended to the target database was used [25], and the best matching sequences from the combined database were indicated by SEQUEST [26]. The searches were done on a cluster of Intel Xeon 80 processors running the Linux operating system. The peptide mass search tolerance was set to 3 Da. No differential modifications were considered. For the aqueous digestion, the mass of the amino acid cysteine was statically modified by +57 Da due to the carboxyamidomethylation of the sample. No enzymatic cleavage conditions were imposed on the database search, so the search space included all candidate peptides whose theoretical mass fell within the 3 Da mass tolerance window, regardless of their tryptic status.

The validity of peptide/spectrum matches was assessed in DTASelect 2 [27] according to the SEQUEST cross-correlation score (XCorr) and the SEQUEST normalized difference in cross-correlation score (DeltaCN). The search results were grouped by charge state (+1, +2, and +3) and tryptic status (fully tryptic, half-tryptic, and non-tryptic), resulting in 9 distinct subgroups. In each of the subgroups, the distribution of XCorr and DeltaCN values for the direct and decoy database hits was obtained, and the two subsets were separated by quadratic discriminant analysis. Outlier points in the two distributions (for example, matches with very low XCorr but very high DeltaCN) were discarded. Full separation of the direct and decoy subsets is not generally possible; therefore, the discriminant score was set such that a false-discovery rate of 5% was determined based on the number of accepted decoy database peptides. This procedure was independently performed on each data subset, resulting in a false-positive rate independent of tryptic status or charge state. In addition, a minimum sequence length of 7 amino-acid residues was required, and each protein on the list was supported by at least two peptide identifications unless specified otherwise. These additional requirements, especially the latter, resulted in the elimination of most decoy database and false-positive hits, as these tended to be overwhelmingly present as proteins identified by single peptide matches. After this last filtering step, the estimated false-discovery rate was reduced to below 1%.

### Selecting differentially expressed proteins with PatternLab's ACFold

ACFold is part of the PatternLab for Proteomics project [13] and considers information from protein fold changes, the AC test [28], and a false-discovery rate (FDR) estimator [29] to pinpoint differentially expressed proteins. We recall that the AC test can be used to calculate the conditional probability of finding a spectral count of *x*_2 _in biological state 2 given that a spectral count of *x*_1 _was observed in biological state 1. The ACFold method was chosen because it is designed to search for differential protein patterns in shotgun proteomic data and can be applied even if the assays are not technical replicates, as in our multi-surfactant shotgun proteomic approach [13,22].

ACFold is effective because drawing conclusions using only a fold change cutoff can shadow information from low-level protein changes that might be important. To account for such, ACFold relies on the AC test to fish out proteins that, despite not having achieved a theoretical optimal fold-cutoff, do nevertheless exhibit a difference in spectral counts between states that is statistically significant. Such proteins are put in evidence to be re-considered in the final analysis or for further experimental validation.

**Figure 1:**
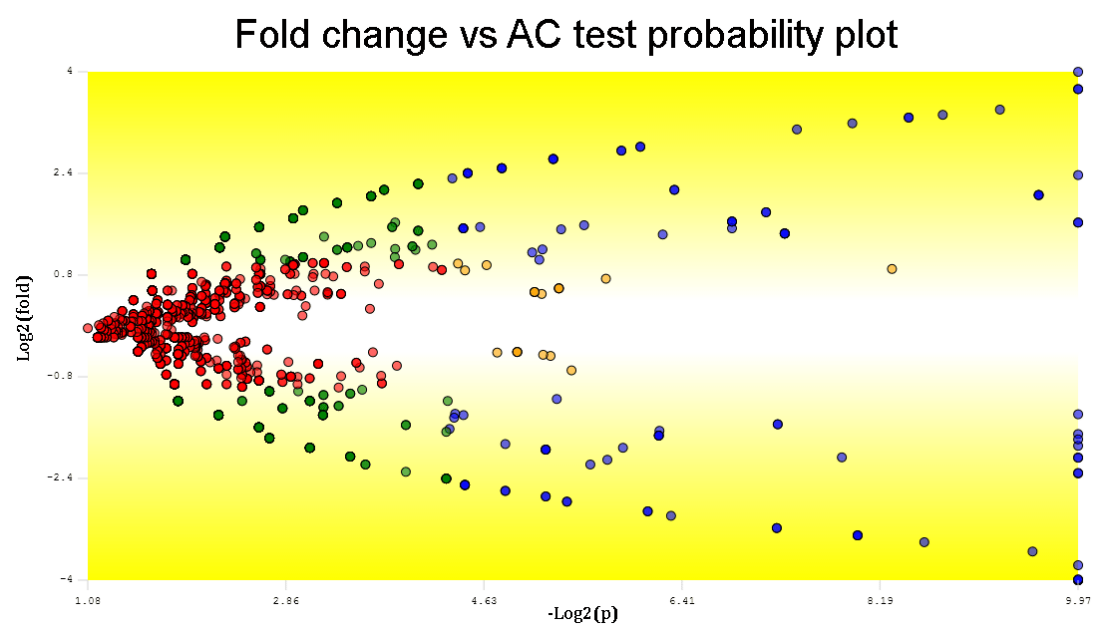
**Fold change versus AC test probability plot**. This plot was obtained using PatternLab's ACFold algorithm and displays the results obtained with the multi-surfactant shotgun proteomic approach when comparing the A172 cell lines before and after the treatment with perillyl alcohol. Each protein (represented as a dot) was mapped according to its log_2_(fold change) on the ordinate (y) axis and -log_2_(1-(AC test *p*-value)) on the abscissa (x) axis. A total of 104 proteins (blue dots) were selected as differentially expressed because they satisfied both the AC test and the FDR *q*-value specified cutoffs. 23 proteins (orange dots) did not meet the fold change cutoff but were indicated as statistically differentially expressed, therefore deserving further analysis. 267 proteins (green dots) met the fold change cutoff, but the AC test indicated that this happened by chance. 2293 proteins (red dots) were pinpointed as not differentially expressed between classes because they failed both the AC test and the fold change cutoffs. The number of dots does not match the number of identified proteins due to the many overlaps.

We refer to Figure 1 to illustrate the output of PatternLab's ACFold graphical user interface and also further details of an ACFold analysis. We also remark that, additionally, PatternLab incorporates the TFold method, in which the t-test substitutes for the AC test, for use when 3 or more replicate readings for each state are available.

In this work, PatternLab's parser was used to convert the DTASelect files from all MudPIT assays into the unified PatternLab format before loading them to the ACFold tool. An FDR *q-*value of 0.1 and an AC test *p*-value of 0.05 were specified. The Row Sigma normalization [13] was chosen for computing the fold changes. The fold change cutoff of 2.5 was empirically specified so as to maximize the number of proteins that satisfy both the FDR and the AC test criteria. We note that higher fold change cutoffs reduce the number of verified hypotheses, usually increasing (decreasing) the number of proteins approved by the FDR (AC test). Finally, a report listing the proteins that satisfied all criteria (ACFoldReport) was exported to text format. This report is also the input to the GOEx analysis. We refer the reader to Figure 1 to illustrate PatternLab's ACFold graphical user interface output of the identified proteins' distribution.

## Implementation

### GO Explorer

GOEx was coded using C# 3.5 and carried a graphical user interface for improved user experience. GOEx requires the downloading of two files: the latest GO ontology (OBO v.1.2 format), freely available at , and the GOA (gene ontology annotation) association file containing the non-redundant, species-specific annotation, freely available at . The latter is necessary to convert the IPI's (international protein indexes), obtained during protein identification, into the GO terms. In this work, we used the gene_ontology_edit.obo (Feb. 08, 2008) and the gene_association.goa_human (Feb. 03, 2008) files. From then on, GOEx parses both files and performs various pre-computations (e.g., mapping all terms descending from a specific term) and associations to speed up the user's experience when analyzing data. All information is then compacted into a binary representation, in a process known as serialization, and saved to disk for quick retrieval during a future use.

Finally, the GOEx panel is unlocked and the GO root terms are listed in the interactive directed acyclic graph (iDAG) interface. The user can then load a report of the differentially expressed proteins (e.g., ACFoldReport) to be analyzed in any of the GOEx study modes: iDAG-driven, specialist-driven, and automatically driven. For convenience, henceforth we refer to the proteins reported in the ACFoldReport as "reported proteins".

### Calculating the over-representation *p*-value

First the accession number listed in the "reported proteins" file are converted into their equivalent GO terms. This conversion entails a mapping that can occur at different levels of the GO hierarchy (not only at the leaves) and sometimes a protein can be mapped onto more than one GO term. While the conversion takes place, tags are maintained for each term indicating which proteins were mapped onto it.

The over-representation *p*-value of term termed as ***S ***relative to the namespace of source (least specific term) ***G ***is computed as follows. Let *g *denote the total number of GO terms in the namespace of source ***G ***and let *s *- 1 be the number of GO terms that descend from ***S***–thus *s *includes ***S ***itself and its descent. The overrepresentation *p-*value of ***S ***must be computed so as to reflect the distinct proteins that were mapped onto the *s *terms. Counting the number of such proteins from the tags maintained during the mapping process is not enough because, in principle, the result may amount to more than *s*. Letting *c*(***S***) be this number of distinct proteins, the count we actually use is then *k *= min{*s*, *c*(***S***)}. The probability of observing these *k *distinct proteins for a randomly selected ***S ***can now be estimated by the hypergeometric distribution: if *X *is the corresponding random variable, then

$$P(X=k)=\frac{\left(\begin{array}{c} t\\ k \end{array}\right)\left(\begin{array}{c} g-t\\ s-k \end{array}\right)}{\left(\begin{array}{c} g\\ s \end{array}\right)},$$

where *t *= min{*g*, *c*(***G***)}, following the same reasoning that led to the definition of *k*. This given, we express the over-representation *p*-value of term ***S ***as the probability of observing *k *or more distinct proteins mapped onto the *s *terms, that is,

$$P(X\geq k)=1-\sum_{i=0}^{k-1}\frac{\left(\begin{array}{c} t\\ i \end{array}\right)\left(\begin{array}{c} g-t\\ s-i \end{array}\right)}{\left(\begin{array}{c} g\\ s \end{array}\right)}.$$

Clearly, the lower this *p*-value the greater the probability mass that lies strictly below *k*.

### Data Analysis

#### a) The GOEx iDAG-driven mode

**Figure 2:**
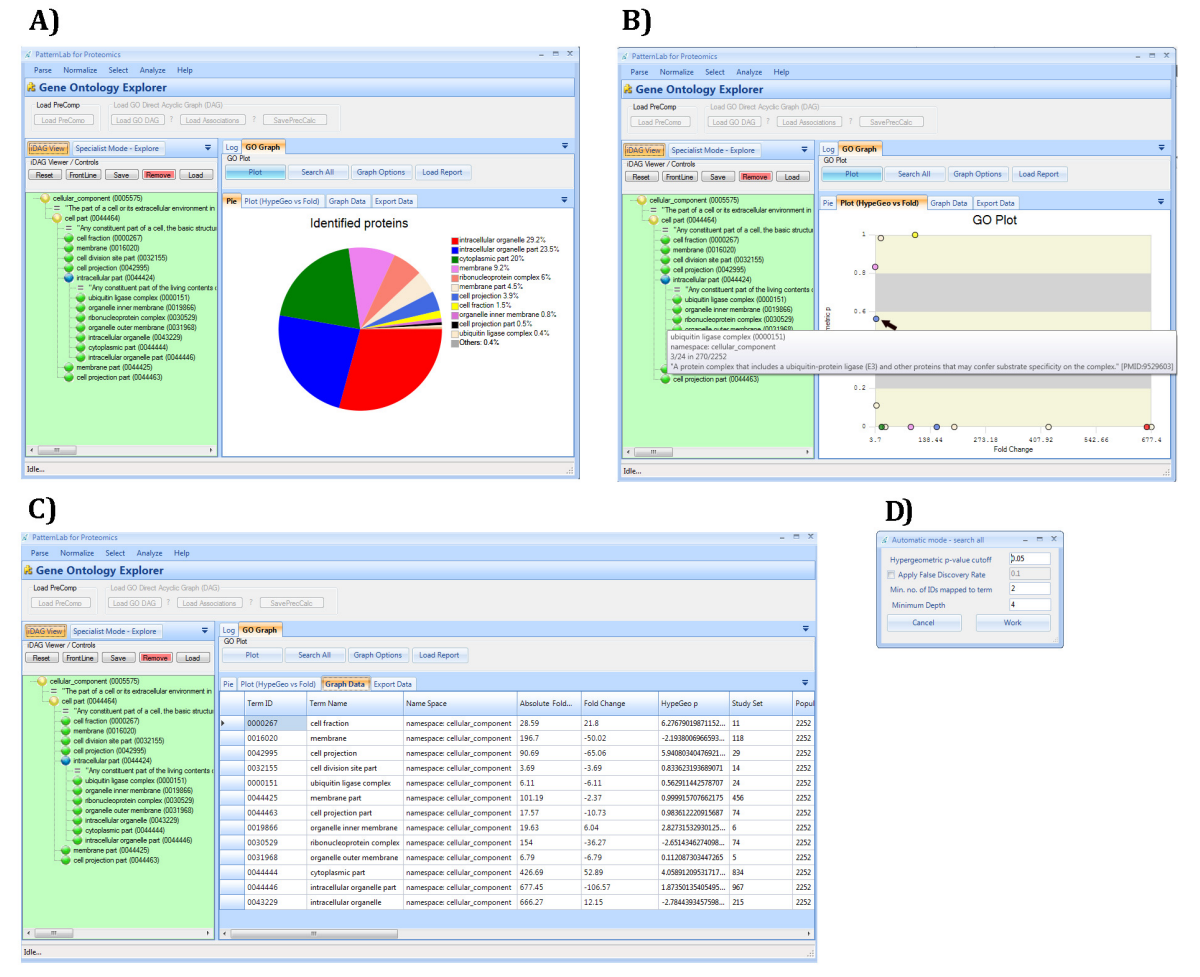
**The GOEx graphical user interface**. A) A pie chart showing the distribution of the identified proteins as mapped onto selected cellular component GO terms is displayed on the right. The level of specificity was chosen according to the iDAG in the left panel. B) The GO terms related to the iDAG terms specified on the left are plotted according to the overrepresentation *p-*value and absolute fold change calculated for them from the identified proteins. The mouse is currently hovering over one term and its GO description is provided in a balloon. A detailed report table can be accessed by clicking on the Graph Data tab. C) Detailed information on the displayed results can be accessed by clicking on the Graph data tab. The table can be dynamically sorted by clicking on the column of interest. A detailed description of each column is addressed in The GOEx report table section. D) The automatic search pop-up window appears when one clicks on the Search all button in the main interface. The user can then select several stringency values to search for statistically overrepresented terms.

This strategy is designed to help guide one's biological questions by leveraging the GO through the iDAG coupled with graphing tools. By clicking on an iDAG term, its child terms appear listed below it; for each child, its over-representation *p*-value (described above) and the sum of the protein fold changes reported for it are computed. Terms having no relation to the reported proteins are automatically deleted to keep the biological questions on track. A "distribution pie chart" (Figure 2A) and a "fold change versus over-representation plot" (Figure 2B) of the displayed iDAG leaf terms are presented. A report table discriminating all calculations and the reported proteins related to each term is also made available. All this information can aid in choosing which term to explore next, if any, thus helping drive one's biological questions. In general, terms having low *p-*values and/or high-magnitude fold changes are good candidates, but there are important exceptions. For example, while exploring for putative molecular functions of our reported proteins, we noted that the "molecular transducer activity" GO term presented a significant fold change (*fold *= 12) but was not statistically over-represented (*p *= 0.97). Even so, by further expanding it and examining its child terms, GOEx revealed the "G-protein coupled receptor activity" term (*fold *= -8, *p *= 0.89) to be associated with our reported proteins, which is only as expected according to previous work related to the effects of POH on tumor cells [21]. This example illustrates that it is possible to draw the important conclusions from fold change data only. GO tools, however, tend to overlook this, being generally limited to taking into account over-representation *p-*values exclusively.

#### b) The GOEx specialist-driven mode

This mode allows an expert to pose questions and retrieve answers in the light of the GO and the reported proteins. For example, it is known that the Ras signaling pathway has a key role in the pathogenesis of GBM by acting as a primary switch that mediates external signals to numerous intracellular signaling pathways [30]. It is also known that POH affects the levels of Ras-related proteins and Ras isoprenylation, thereby altering cellular physiology [31]. Entering the key word "Ras" to the search facility of the GOEx specialist-driven mode produced, in a log file, a list of all GO terms containing the key word in their names or descriptions. Terms related to the reported proteins (either through fold change or over-representation) were analyzed, plotted, and added to the report table. The result pointed to the "Ras protein signal transduction" term as being related to our dataset despite not quite qualifying as statistically over-represented (*p *= 0.06 against a *p*-value cutoff of 0.05). This example indicates that, even though a term's over-representation may not be indisputably significant (and thus the term might not be detected during an automatic search, as in most GO tools), that term may nevertheless embody the correct answer. In the case at hand, the literature gives plenty of supporting evidence to corroborate the hypothesis of alterations in the Ras pathway. This is further addressed in the Results and discussion section.

#### c) The GOEx automatic mode

The automatic mode (search all) performs an extensive analysis by searching for relations between the reported proteins and each and every GO term. This method requires the user to specify the desired minimum number of proteins related to a GO term, a minimum GO depth, and an over-representation *p*-value and optionally a false-discovery rate [29]) cutoff. We define GO depth as the shortest path from a term to its root. From then on, GOEx will evaluate all GO terms. The ones bearing relation to the reported proteins will be listed in the report table (described in section d) and plotted in the "distribution pie chart" and "fold change versus over-representation plot". This mode is optimized for multi-core processors and relies on concurrent computation to speed up its task.

#### d) The GOEx report table

All GOEx query methods provide the already mentioned complementary report table that can be dynamically sorted according to convenience. The table headers include: GO ID, Term Name, Namespace, Absolute Fold Change, Fold Change, HypeGeo P, Study Set, Population, Identified in Study Set, Identified in Population, Proteins IPI's and Folds, GO depth, and Description. GO ID and Term Name specifies the unique GO identifier and its name as given in the GO. Namespace points to which GO namespace the selected term belongs to (molecular function, cellular component, or biological process). Absolute Fold Change is the sum of the absolute values of the fold changes of all proteins mapped onto a given GO term. Similarly, Fold Change is the sum all their fold change values. Current gene ontology tools usually do not report fold change information. HypeGeo P is an abbreviation for the term's over-representation *p*-value. Study Set refers to all the terms that descend from a given term. Population stands for all the terms contained within the specified term's namespace. Identified Proteins indicates how many of the proteins discriminated in the ACFoldReport were mapped onto the specified term. Proteins IPI's and Folds discriminates all the proteins, and respective fold changes, mapped onto the selected term. Finally, Description refers to the term's GO description.

## Results and discussion

**Figure 3:**
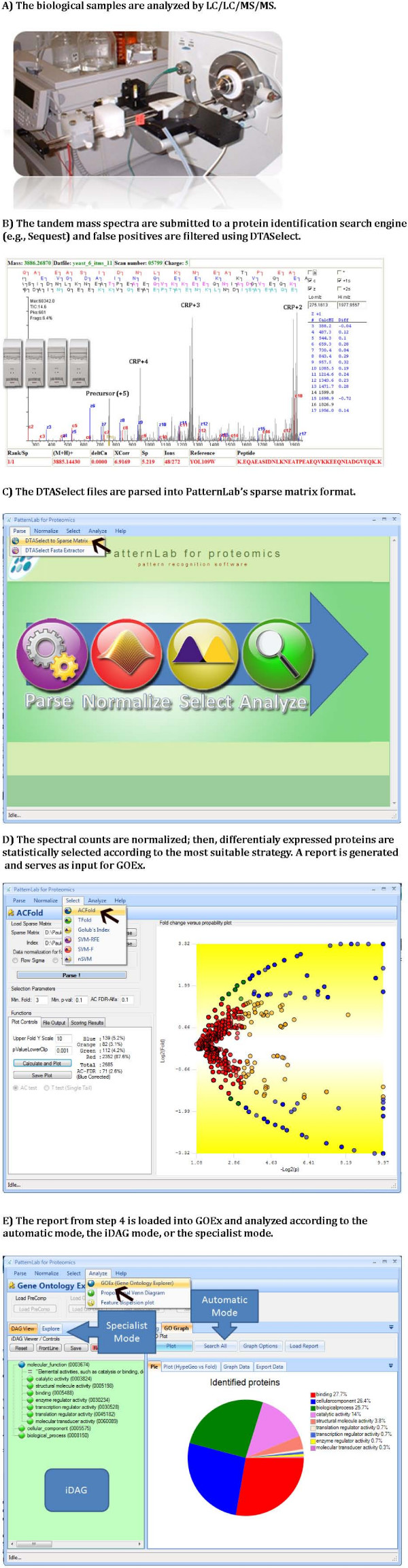
**Workflow**. Key steps in the workflow, ranging from the mass spectral acquisition to the final GOEx analysis.

We refer the reader to Figure 3 for an illustration of the main steps that led to the results we now present.

### Protein identification by the multi-surfactant shotgun proteomic approach

Protein solubility varies in different buffers and in the presence of different types of detergents. Therefore, protein solubilization by different MS-compatible detergents can provide complementary data [22]. In this way, our multi-surfactant proteomic approach can potentially cover a larger portion of the proteome than the traditional technical replicate approach, and improve the GO analysis [22]. Our proteomic methodology identified a total of 2687 proteins during all six MudPIT runs and PatternLab's ACFold selected 104 of them as differentially expressed. An additional 23 proteins that did not satisfy our fold cutoff but had a very low AC test *p*-value (the ACFold orange group) were independently evaluated and included in our list.

As far as we know, our A172-POH dataset is the largest one concerning GBM A172 cells. Such repository, together with the DTASelect files and the reported differentially expressed proteins, is available for download at the PatternLab for Proteomics project website and can be a valuable source to test future GO approaches. Taken together, the proteins identified in the present study can also provide important fundamental information about the cellular response to POH treatment.

### The GOEx specialist mode results

The "Ras protein signal transduction" term was linked to two proteins: transforming protein RhoA (IPI00478231) and Rho-related GTP-binding protein RhoB (IPI00000041). RhoA is involved in regulating the signal transduction pathway between the plasma membrane receptors for the assembly of focal adhesions and actin stress fibers. Yan and collaborators have reported RhoA's expression to positively correlate with the degree of malignancy in astrocytomas and that its expression is increased in various neoplasias. The authors also suggest important implications of RhoA in both the clinical prognosis and the biology of these neoplasms, and even suggest using it as a prognostic biomarker [32]. Our results showed a down-regulation of ~3× for RhoA after the POH treatment, showing POH to be effective as a chemotherapeutic agent.

RhoB was also down-regulated (~4×) after the POH treatment. RhoB is linked with endothelial cell survival during angiogenesis and has been hypothesized to have a role in TNFalpha-induced angiogenesis through the regulation of Akt activation, being therefore important for tissue repair during acute inflammatory responses [33]. Thus, the fact that POH is an angiogenesis inhibitor is in agreement with our results [34]. Moreover, the authors also report that inhibiting the farnesylation of RhoB is a strategy for treatment. Indeed, one of the key effects of POH is to inhibit the farnesylation of Ras proteins, preventing them from docking in the plasma membrane and initiating signal transduction [21].

### The GOEx automatic search result

**Figure 4:**
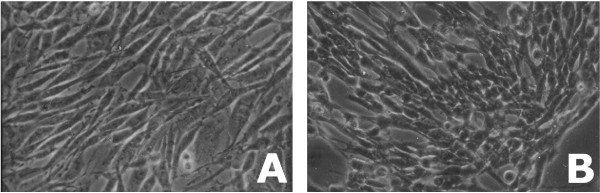
**Microscopy images of the A172 cells**. These microscopy images (200×) show the A172 cell line before (A) and after treatment with POH during 1.5 h. The cellular morphology changes and the cells become rounder after the POH treatment.

The GOEx automatic mode can provide complementary results to the specialist when compared to the iDAG-driven mode, as exemplified in the Implementation section. We performed an automatic search on our dataset using an FDR of 0.05 and eliminating terms that had each only one protein assigned to it. The results pointed mostly to terms related to cell cycle, alcohol catabolism, the Ras pathway, apoptosis, and stress response. Examples of terms belonging to the molecular function namespace and selected as overrepresented include, but are not limited to: purine nucleotide binding, hydrolase activity–acting on acid anhydrides–in phosphorus-containing anhydrides, structural molecule activity, and cytoskeletal protein binding. Similarly, terms belonging to the cellular component namespace include, but are not limited to: membrane-bound vesicle, actin filament bundle, cytoskeletal part, and cytosolic part. Finally, terms from the biological process namespace include, but are not limited to: microtubule-based process, actin filament-based process, regulation of apoptosis, alcohol metabolic process, and GTP metabolic process. Indeed, apoptosis, changes in morphology, and most of the terms listed were only expected, according to previous work [16,20,21]; microscopy images of the cells can be found in Figure 4.

### The GOEx methodology

There are several methods to compute an over-representation *p*-value; examples are: the hypergeometric [35], binomial, *χ*^2 ^(chi-square), and Fisher's exact [36] tests; their differences have been reported not to be dramatic for the GO overrepresentation problem [37]. Most GO-based tools are limited to what is equivalent to the GOEx automatic search in terms of limiting the search to finding statistically over-represented terms. To speed up their analyses, they usually do not offer over-representation calculation using the hypergeometric distribution and/or use GO-slim, a reduced version of GO. However, analyses according to the latter are restricted to the higher GO levels, which contrast sharply with our approach, which takes into account all levels and every term. This limitation could lead to missing differences that are detectable only at more refined levels. With the advent of faster microprocessors, the time to complete a full GO search has dramatically decreased, so what was once considered an issue to worry about has been downshifted. Nevertheless, GOEx also takes advantage of the new multi-core chips to perform concurrent computing to accelerate the automatic search.

Even though variations on how to find over-represented terms can be proposed, there is no reference standard on how to properly measure the gains. So comparisons between methods are bound, to some extent, to be disputed [38]. GOEx stands out among other methods because it lies within a complete workflow to analyze shotgun proteomic experiments that rely on spectral counting. Most importantly, its reports combine information from fold changes with statistics. As we exemplified, these two types of information are complementary, yet most existing GO tools do not take this fact into account. In any given biological phenomenon, different genes are regulated to different extents. The data providing information about differential protein expression can be useful in assigning different weights to the corresponding biological processes involved and aid in inferring which biological process is more relevant [37]. Certainly, the greatest limitation of GOEx, and of all existing GO-based tools as well, is that GO, the IPI database, and the mappings, all of which serve as foundations for such tools, are not complete, which evidently affects the results they yield. Such limitation is inevitable but tends to become less important as these databases are expanded.

In all, GOEx provides several strategies to explore how the proteins of interest are distributed among GO terms. Differently than the automatically driven methods of previous software, GOEx embodies flexible exploratory tools. For example, as terms are expanded in the iDAG, child terms onto which any identified protein is mapped are kept even if not statistically enriched. This retains terms that could contain a single protein and yet be crucial for drawing conclusions. Thus, GOEx's iDAG or specialist mode can determine both whether GO categories are statistically over-represented and whether there are significant changes for individual proteins.

It seems to be a consensus that web-based tools are more liable because the researcher can be assured to be using the software's latest version: maintaining a stand-alone installation represents one more chore to the user. However, the GOEx installation has been designed to be straightforward; in fact, it can be done with one single click of the mouse. If the application needs upgrades or detects any missing components, they are automatically downloaded. Nevertheless, if a major change has been deployed but the user is unsatisfied, a rollback (restore) can be done in one single step, differently than the web-based case, in which one is forced to use the available version. In this way, GOEx provides benefits in a locally installed distribution, besides not forcing the user to share sensitive data with an unknown and remote server. In conclusion, GOEx facilitates organism-specific searches using GO through a rich graphical user interface. It is a useful, friendly, and simple to use tool, specialized for biologists who wish to analyze spectral counting data from shotgun proteomics.

## Availability and requirements

GOEx is available for download at  and is free for academic use. It was programmed in C# and requires .NET 3.5 framework (can be automatically installed) and a windows (VISTA or XP) personal computer.

## Competing interests

The authors declare that they have no competing interests.

## Authors' contributions

PCC coded the software and wrote the first draft of the manuscript under the guidance of VCB and JRY. EIC and JSGF generated the MudPIT experimental data, prepared the POH-A172 cells, helped test the software, and suggested the inclusion of important features. WMD discussed several aspects of the software. GBD and MGCC participated during all phases as JSGF's doctoral advisers. All authors read and approved the final manuscript.
